# NPDBEjeCol: A Natural Products Database from Colombia

**DOI:** 10.1021/acsomega.5c00936

**Published:** 2025-02-27

**Authors:** Johny R. Rodríguez-Pérez, Hoover A. Valencia-Sánchez, Oscar M. Mosquera-Martínez, Alejandro Gómez-Garcia, José L. Medina-Franco, Héctor F. Cortes-Hernández

**Affiliations:** † GIFAMol Research Group, School of Chemistry Technology, Universidad Tecnológica de Pereira, Pereira 660003, Colombia; ‡ GBPN Research Group, School of Chemistry Technology, Universidad Tecnológica de Pereira, Pereira 660003, Colombia; § DIFACQUIM Research Group, Department of Pharmacy, School of Chemistry, Universidad Nacional Autónoma de México, Avenida Universidad 3000, Mexico City 04510, Mexico

## Abstract

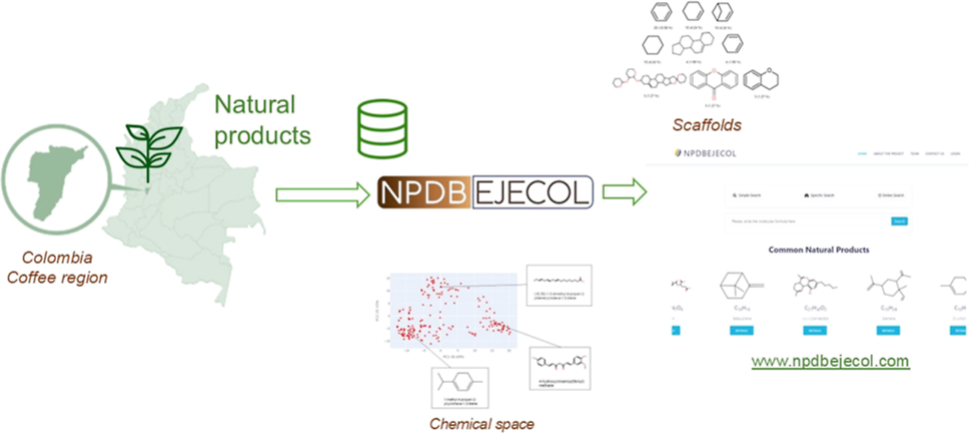

The aim of this research is to introduce the first curated natural product database from Colombia, Natural Products DataBase EjeCol (NPDBEjeCol), that has been made publicly available at www.npdbejecol.com. The compound library, compiled from the peer-reviewed literature, is composed of natural products derived from plants in the *coffee region* of Colombia. After extensive data standardization and curation, molecular descriptors of pharmaceutical relevance and molecular fingerprints of different designs were calculated in order to evaluate the structural diversity and explore their chemical space of compounds in NPDBEjeCol in comparison with natural products reference libraries. The current version of NPDBEjeCol contains 236 molecules, for which detailed information is available. This includes the compound name, linear notation, references to the peer-reviewed literature, CAS number, synonym names, and constitutional descriptors. Analysis of the drug-like properties suggest that NPDBEjeCol natural products are on average, compliant with the empirical Lipinski’s rule. Visualizations of the chemical space based on fingerprints uncovered one to three clusters of compounds and fragments. Among the phytochemical groups present in the database, terpenes are the most prominent, particularly those derived from monoterpenes and sesquiterpenes. NPDBEjeCol is the first Colombian natural products database of its kind in the country that can be publicly accessed through a web portal, facilitating open query, navigation, and visualization of the identified molecules.

## 1 Introduction

Historically, natural products have been identified as the primary source of compounds utilized in the development of pharmaceuticals, cosmetics, and food products. Currently, these remain an important resource for technological and socio economic development.^1^ In the field of chemistry, natural products are the subject of considerable interest from the scientific community due to their abundance and relevance in ecology, phytochemistry, medicinal chemistry, and molecular biology.^2^ Furthermore, these compounds and their semisynthetic derivatives represent an important source of drug candidates for a wide range of diseases, making them attractive as bioactive compounds susceptible to further development or optimization, and as substrates with unique substructures.^3^ Colombia has a significant biodiversity. In terms of plants, the country has the second-highest diversity in the world, with more than 28,000 identified species. These represent at least 10% of the total number that inhabit the planet, with about 15% still to be discovered.^4^ In Colombia, 2404 species of medicinal plants have been identified, of which 1656 are native neotropical species that grow in the country, 214 are considered endemic to Colombia due to their current distribution, probably originating either from Colombia or from other Neotropical countries.^5^ The Colombian Vademecum of Medicinal Plants included 133 accepted species of plants with therapeutic value,^6^ the majority of which are introduced and therefore not native.^7^

At the present time, in Colombia, information related to natural products derived from plants has not been organized or compiled. The Intergovernmental Science-Policy Platform on Biodiversity and Ecosystem Services (IPBES), a collaborator entity with the United Nations Environment Programme (UNEP), serves as an intermediary between the scientific community and policy-making institutions. It proposes as a conservation strategy the compilation of peer-reviewed works on a range of scientific topics into databases or multidisciplinary platforms as a conservation strategy (Pilon et al., 2017). Therefore, databases of diverse types have served as pivotal hubs for the acquisition, organization, and distribution of information aimed at addressing issues related to human and environmental concerns. Such databases are particularly useful in developing multidisciplinary research fields, including medicinal chemistry, cheminformatics, ethnopharmacology, and omics approaches.^1^ Examples of these resources include Protein Data Bank (PDB), GenBank, Databank of Japan, Peptide Atlas, Global Natural Product Social Molecular Networking, PubChem, ChemSpider, ChemBank, ChEMBL, and DrugBank, among others.

Concerning compound databases that are specifically oriented toward natural products, it should be noted that they are not specialized in any particular type of natural product. Instead, they are presented as catalogs for various purposes, including in silico detection of activity prediction and molecular docking.^8^ Notable examples include Collection of Open Natural Products database (COCONUT), SuperNatural II, Universal Natural Products Database (UNPD), Natural Product Activity and Species Source Database (NPASS), ZINC, RIKEN Natural Products Encyclopedia (NPEdia), a three-dimensional-structure database of natural metabolites (3DMET), the Chinese Natural Products Database (CNPD), and several others. In 2020, Sorokina et al. conducted an inventory of more than 120 natural product-related databases published between 2000 and 2019.^8^ Their findings revealed that 16% of the databases are not available online, 40% are commercial, and the remainder are freely accessible. Latin America shows at least one-third of the global biodiversity. Several countries in the region are considered a megadiverse: Bolivia, Brazil, Colombia, Costa Rica, Ecuador, Mexico, Peru, and Venezuela.^9^ Databases containing natural products (NP) from some Latin American countries have been published in recent years, NaturAr^10^ (Argentina), NuBBEDB,^1^ SistematX,^11^ UEFS^12^ (Brazil), CIFPMA^13^ (Panama), PeruNPDB^14^ (Peru), UNIIQUIM,^15^ and BIOFACQUIM^16^ (Mexico). Currently, there is an initiative to compile the natural product databases of Latin America into a single database designed Latin American Natural Product Database (LaNAPDB).^17^ These molecule libraries are employed extensively in cheminformatics studies, particularly for the identification of drug candidates. They serve an important role in the pharmaceutical industry as sources of new medicines or drug candidates that can be used for subsequent optimization.

At the time of writing, there is currently no online database in Colombia available that presents natural products derived from plants. This study represents a significant initial step toward the development and implementation of a database of natural products focused on a specific region of the country. The Colombian *coffee region* is a geographical area in Colombia renowned for the high quality of its coffee production. Situated at the center of the Colombian Andes, the region encompasses the departments (similar to states in other countries) of Caldas, Quindío, Risaralda, and the northwest of Tolima. The region is distinguished by its mountainous topography and volcanic soils. The altitudes in this area range between 1200 and 2000 m above sea level, resulting in a significant diversity of microclimates and, consequently, a vast array of plant species.^18^

The purpose of developing a database of natural products derived from plants in the *coffee region* of Colombia is to identify and organize, in a single repository, the research conducted in recent years on isolated and characterized natural products from plants studied in this region. The database, which is freely available, will provide a valuable resource for researchers, offering a convenient platform for data consultation and a Web site that provides data and information to support natural product research and various cheminformatics studies including virtual screening. Moreover, it is expected that the newly introduced database, the so-called NPDBEjeCol, will be an initiative to incorporate more studies from other regions within the country over time. Additionally, the database will facilitate the inclusion of natural products derived from sources other than plants in Colombia.

## 2 Materials and Methods

### 2.1 NPDBEjeCol Database

The database of natural products derived from plants in the *coffee region* of Colombia was constructed through a bibliographic search, with other natural product collections such as NuBBE_DB_,^1^ BIOFACQUIM,^16^ and PeruNPDB^14^ as references. The first version of NPDBEjeCol was derived from a comprehensive search of multiple databases. SCOPUS, Web Of Science, and Google Scholar were utilized, employing bibliometric equations that incorporated relevant terms. The following search terms were used: “*natural products*”, “*secondary metabolites*”, “*Colombia*”, “*Quindío*”, “*Risaralda*”, “*Caldas*”, “*Tolima*”, “*molecule*”, “*elucidation*”, and “*characterization*”. The search yielded lists of documents that were reviewed to identify research meeting the following criteria: (1) studies that led to the identification of natural products, (2) those where such molecules were obtained from plants, and (3) cases where the collection of the plants was conducted in the *coffee region* of Colombia. Additionally, the research groups that have worked on natural products, particularly the Biotechnology and Natural Products Group of the Technological University of Pereira (GBPN), provided further information. It should be noted that this is the first version of NPDBEjeCol, and future versions will expand the geographical search and include other sources of validated information. The long-term goal is to construct a comprehensive database of Colombian natural products derived from biodiversity.

### 2.2 Data Set Standardization

The chemical structures of the natural products identified in the literature search described in Section 2.1 were encoded as SMILES strings (Simplified Molecular Input Line Entry System).^19^ To achieve this, the information for this line notation was searched for and retrieved using the public server PubChem.^20^ The data set was standardized using the open source chemoinformatics toolkit RDKit^21^ and MolVS,^22^ using a protocol described by Sánchez-Cruz et al.^23^ The entire process was performed using the Standardizer, LargestFragmentChooser, Uncharger, Reionizer, and TautomerCanonicalizer functions implemented in the MolVS molecule validation and standardization tool for the open-source chemoinformatics toolkit RDKit. The chemical compounds were evaluated according to several criteria, including the presence of specific chemical elements, such as H, B, C, N, O, F, Si, P, S, Cl, Se, Br, and I. Stereochemistry information, as indicated in the original sources, was preserved. In cases where an entry consisted of multiple components, the largest chemical structure was retained. The remaining molecules were neutralized and reionized to generate the corresponding canonical tautomer. Finally, duplicated compounds were removed.

### 2.3 Molecular Properties

The NPDBEjeCol curated database was characterized by calculating and analyzing the distribution of molecular descriptors of pharmaceutical interest. These included molecular weight (MW), number of hydrogen bond acceptors (HBA), number of hydrogen bond donors (HBD), partition coefficient (logP), and topological polar surface area (TPSA). Additional descriptors such as the fraction of carbon sp^3^ atoms (FCSP3), number of rings (NumRings), number of heteroatoms (HetAtoms), and the number of rotatable bonds (RotBonds) were calculated. The aforementioned properties were calculated using the RDKit toolkit. Averages of the distributions were compared to the published values of other natural product databases.

### 2.4 Scaffold Content

The analysis of scaffold content facilitated the identification of the most common scaffolds in composite data sets. The predominant core molecular scaffolds in NPDBEjeCol were identified using the definition proposed by Bemis and Murcko,^24^ wherein the core scaffold is derived by systematically removing compound side chains. Subsequently, the prevalent scaffolds in NPDBEjeCol were then compared with the reported identity and fraction of scaffolds in the literature for other compound databases.

### 2.5 Fragments Diversity

The compounds and molecular fragments were analyzed. Molecular fragments were generated using the REtrosyntheric Combinatorial Analysis Procedure (RECAP) implemented in the RDKit toolkit. The RECAP algorithm is based on the cleavage of 11 synthetic rules.^25^ In order to ensure the reliability of the results, compounds with a MW above 1000 Da were excluded from the fragmentation process.

### 2.6 Fingerprint-Based Diversity

The structural diversity of compounds and fragments was evaluated through the calculation of pairwise similarity values calculated with the Tanimoto coefficient,^26^ for radius 2 Morgan fingerprints (Morgan2, 1024 bits)^27^ and Molecular ACCes System (MACCS) keys (166 bits).^28^

### 2.7 Visual Representation of Chemical Space

The chemical space has been defined as a Cartesian space of dimension M, wherein each dimension represents descriptors or properties that encode a molecule. The length of the descriptor sets determines the number of dimensions of each chemical space.^29^ Two-dimensional reduction techniques were used to generate a visual representation of the chemical space: t-distributed stochastic neighbor embedding (t-SNE)^30^ and principal component analysis (PCA).^31^ Similarity matrices for PCA were calculated using the Tanimoto coefficient with MACCS keys and Morgan2 fingerprints. The t-SNE analysis and PCA were performed by modifying a Python script that had been developed by the DIFACQUIM research group.^32^

### 2.8 Commercial Availability of Natural Products from NPDBEjeCol

The commercial availability of NPDBEjeCol natural products was evaluated using information available in the PubChem database^33^ as of June 2024.

### 2.9 NPDBEjeCol Web Design

For the web design of the database and its online functionality, the graphical interfaces of other natural products databases were taken as reference, including: COCONUT, BIOFACQUIM, PERUNPDB and NuBBE_DB_. Based on these parameters, the minimum criteria for web design were established. To facilitate the databasés development, a client-server infrastructure was implemented. This comprised an Apache web services server, a database engine, and the PHP language, all of which were configured on the server.

## 3 Results and Discussion

### 3.1 NPDBEjeCol Database

A systematic search of the literature was conducted using bibliometric equations with the terms indicated in Section 2.1. The results were limited to articles published up to December 2023. The second filter entailed a detailed examination of the information, further refining the search, and verifying that a natural product was identified, and confirming that it originated from a plant collected in the *coffee region* of Colombia. Following the application of the filters, a total of 16 documents were found: 12 scientific articles and four theses. This was preceded by a bibliometric review of the current state of research on natural products from plants in the Colombian *coffee region*, which will be presented in a peer-reviewed research article.^34^ It is noteworthy that this manuscript discloses the first version of NPDBEjeCol as a proof-of-concept collection, which is focused to the *coffee region* of Colombia. At the time of writing, no other databases of natural product molecules have been published for Colombia. It is anticipated that future versions will update the database content by increasing the number of entries, geographical coverage, and sources of natural products beyond plants.

The current version of NPDBEjeCol contains the following information for each compound: identification number, compound name, SMILES strings, reference (including journal name, Digital Object Identifier (DOI) number, and year of publication), CAS number, synonym names, and constitutional descriptors.

### 3.2 Data Set Standardization

Following the standardization of the data set, any duplicate molecules reported by more than one source of information were removed, reducing the number of molecules from 306 to 236. The application of the standardization protocol ensured the preservation of stereochemistry of the molecules when reported and their presentation in their neutral forms. A canonical SMILES representation was thus generated. Duplicate structures were removed. The first version of NPDBEjeCol contains a total of 236 distinct molecules derived from the plants studied in the *coffee region* of Colombia, following standardization.

### 3.3 Molecular Properties

**Figure 1:**
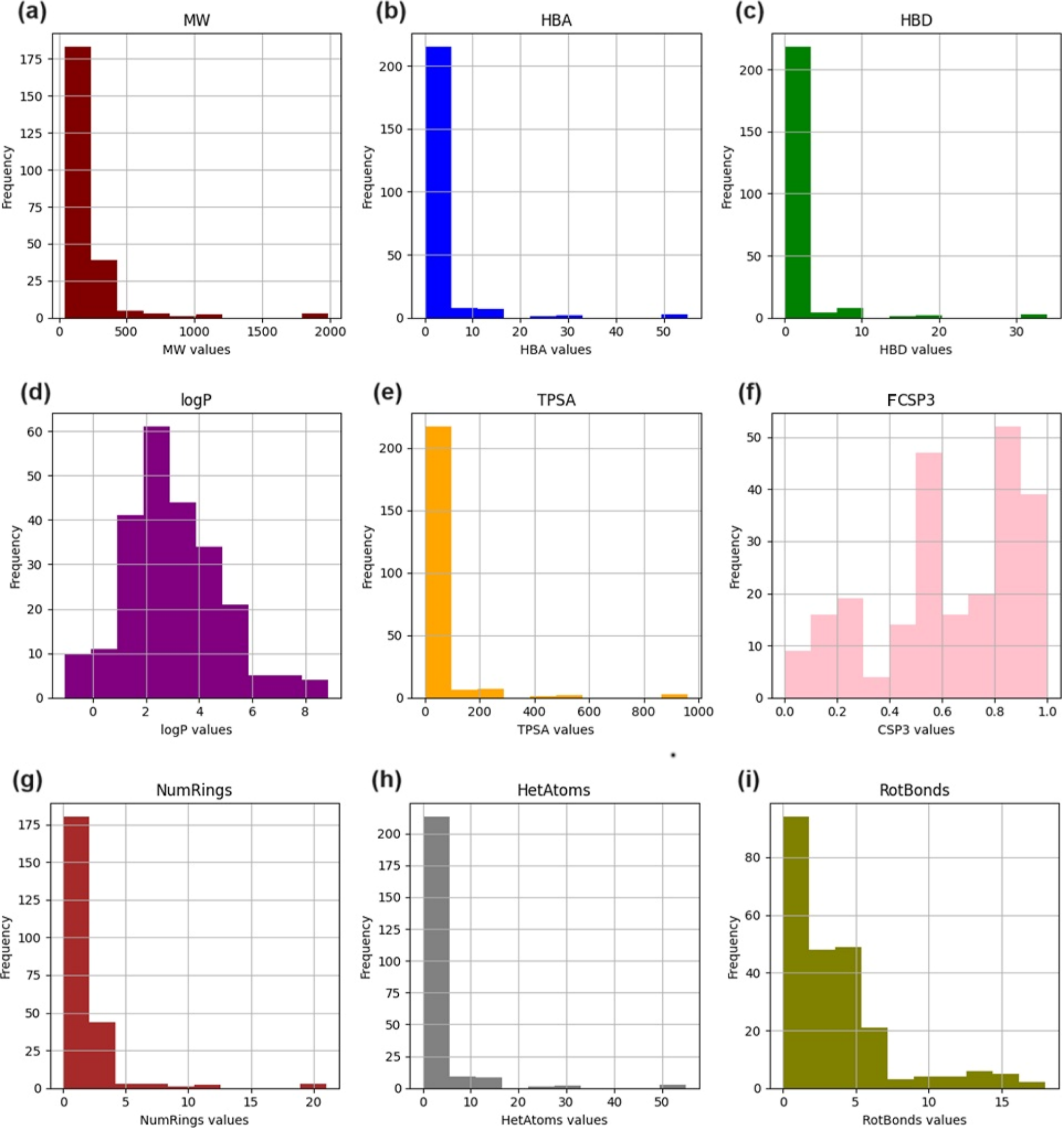
Distribution charts for molecular descriptors of NPDBEjeCol. (a) MW: molecular weight; (b) HBA: number of H-bond acceptor atoms; (c) HBD: number of H-bond donor atoms; (d) log *P*: octanol/water partition coefficient; (e) TPSA: topological surface area; (f) FCSP3: fraction of sp^3^ carbon atoms; (g) NumRings: number of rings; (h) HetAtoms: number of heteroatoms; (i) RotBonds: number of rotatable bonds.

The molecular properties of all 236 compounds in NPDBEjeCol were calculated. The distribution of each property is shown as histograms in Figure 1. It is highlighted that most of the NPDBEjeCol molecules have a MW of less than 500 g/mol, with an average of 234.77 g/mol. This average is lower than that of other databases, including BIOFACQUIM, which is between 340.5 and 412 g/mol,^16^ and NUBBE_DB_, which is 386.3 g/mol.^35^ The number of HBA atoms is, on average, three, which is lower than the average of four observed in BIOFACQUIM and NUBBE_DB_. The number of HBD atoms is one, which is identical to the value reported by BIOFACQUIM and NUBBE_DB_.^16^ The log *P* is 3.02 on average, with values ranging from 0.19 to 8.84, making the average an intermediate value between that reported for BIOFACQUIM (2.8) and that reported for NUBBE_DB_ (3.4).^16^ Of note, to compare NPDBEjeCol with the reference databases, BIOFACQUIM and NUBBE_DB_ the same protocol was used to curate and standardize the three compound databases. These properties can be related to Lipinski’s Rule of Five, which is a set of guidelines for predicting the oral bioavailability of a compound.^36^ When interpreting the properties evaluated in Lipinski’s rule of five, a compound is more likely to be orally bioavailable; if it has a MW less than 500 g/mol, no more than five HBD, no more than ten HBA, and a lower log *P* value to five. On average, the molecules in NPDBEjeCol would be considered to fall within these parameters.

About the TPSA average is 47.72, which is lower than the averages reported for BIOFACQUIM and NUBBE_DB_, which are 71.1 and 64.6, respectively.^16^ Additionally, other values found, including an average FCSP3 of 0.62, a mean of less than two for the number of rings per molecule, approximately three for number of heteroatoms, and three for the average number of rotatable bonds. These properties are significant in the context of drug discovery, as they influence the drug-likeness and pharmacokinetic properties of the compounds.^37^ Lower TPSA values, fewer rings, and a moderate number of rotatable bonds can enhance a molecule’s ability to permeate cell membranes, making them more likely to be successful as drug candidates.^38^Table 1 presents a summary of the calculated measures of central tendency for the descriptors. It is noteworthy that the mean number of carbons in the NPDBEjeCol molecules is approximately 14, indicating the presence of small molecules, however the standard deviation are close to 11 this shows that there is a wide dispersion variation in the number of atoms that occur in the molecules, the value of the median is 11. These molecules exhibit an average of three oxygens and a minimal amount of nitrogen, with an average of 0.1 atom per molecule. Other molecular databases with comparable information present larger molecules, with an average of 25.6 carbon atoms in COCONUT, 26.5 in FooDB, and 18.05 in Dark Chemical Matter Library (DCM). With regard to the number of oxygens and nitrogens found in databases, the respective values were 6.16 and 1.44 for COCONUT, 7.34 and 0.66 for FooDB, 3.25 and 2.85 for DCM.^3^ In the case of the standard deviation of the number of oxygens, the value is significantly higher than the mean, indicating a large variability in the number of these atoms in the molecules. Similarly, the number of nitrogens also shows a standard deviation higher than the mean. The median for the number of nitrogens is zero, which represents the central value for this type of atom. These data reflect the structural diversity of the molecules in terms of the number of atoms present.

**Table 1:** Structural Composition of Compound Library NPDBEjeCol

molecular descriptor	mean	standard deviation	median
number carbons	13.96	10.54	11.0
number oxygens	2.78	6.83	1.0
number nitrogens	0.12	0.68	0.0
fraction of carbons	0.87	0.12	0.9
fraction of oxygens	0.12	0.11	0.09
fraction of nitrogens	0.01	0.04	0.0
fraction of carbon sp^3^	0.63	0.28	0.67
fraction of chiral carbons	0.09	0.12	0.0
molecular weight	234.77	234.43	183.16
number heavy atoms	16.88	16.81	13.0
rings	1.69	2.8	1.0
aliphatic rings	1.11	1.82	0.0
aromatic rings	0.58	1.45	0.0
heterocycles	0.45	1.47	0.0
aliphatic heterocycles	0.36	1.43	0.0
aromatic heterocycles	0.58	1.45	0.0
spiro atoms	0.02	0.14	0.0
bridgehead atoms	0.41	1.6	0.0

### 3.4 Relevant Phytochemical Groups

The phytochemical groups present in the 236 NPDBEjeCol molecules include monoterpenoid derivatives, which are compounds formed by two isoprene units (10 carbon atoms), sesquiterpenoids, compounds derived from sesquiterpenes (formed by three isoprene units, 15 carbon atoms), and fatty esters, compounds that are formed when a fatty acid reacts with an alcohol, in this case of vegetable origin, being present in essential oils. Fatty acids and conjugates are carboxylic acids with long chains of carbon atoms, which can be saturated or unsaturated. Phenolic acids C6–C1 (compounds containing a benzene ring linked to a hydroxyl group –OH and a carboxyl group –COOH), phenolic acids of type C6–C1 have a basic structure of a phenol group (C6) linked to a methylene group (–CH_2_–) which is linked to a carboxyl group. To a lesser extent, there are natural products derived from steroids (also known as phytosterols if they are derived from plants, they are lipid compounds that have a chemical structure similar to that of cholesterol), meroterpenoids (hybrid compounds that combine parts of terpenes with other functional groups such as phenols, organic acids, among others, fatty acids), flavonoids (phenolic compounds containing a C6–C3–C6 flavono nucleus), phenylpropanoids (C6–C3), pseudoalkaloids (compounds that, although structurally similar to alkaloids, are not derived from amino acids, but from other biosynthetic sources). In addition to the previously mentioned compounds, the following phenolic compounds are also present: diarylheptanoids, which are phenolic compounds formed by two units of C6 benzene rings linked by a chain of seven carbon atoms, and C6–C1 phenolic acids, compounds derived from phenylpropane, which has a basic structure of C6 (benzene ring) and C3 (three-carbon side chain). In smaller quantities, xanthones are present. These are phenolic compounds derived from the chemical structure of xanthans, which are a class of heterocyclic compounds characterized by their structure of benzene rings fused with an oxygen ring.

### 3.5 Scaffold Content

**Figure 2:**
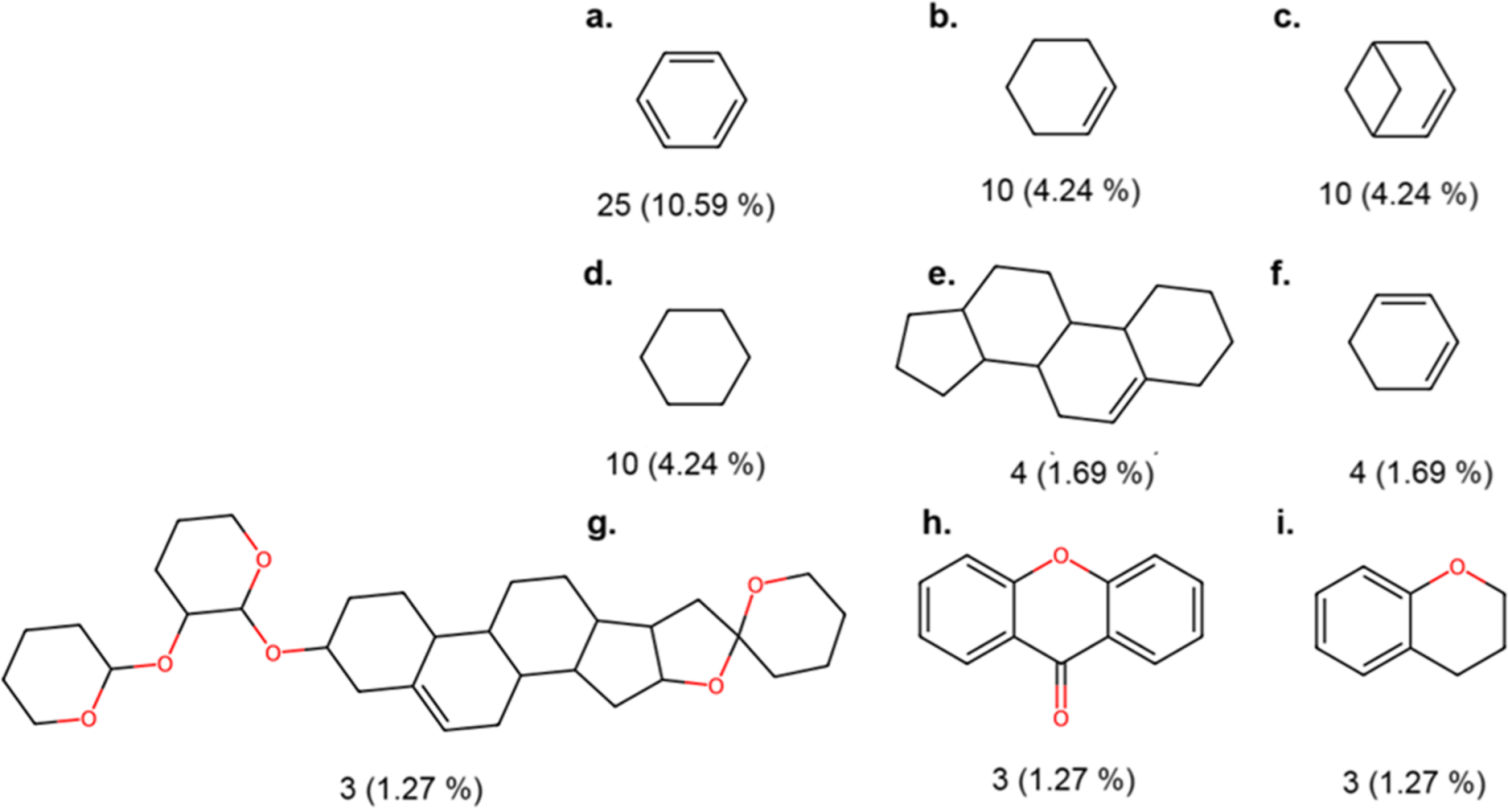
Most frequent scaffolds with at least one ring from NPDBEjeCol. The percentage of each scaffold within its database is indicated below the chemical structures.

Most of the molecules in NPDBEjeCol are linear scaffolds (no rings), comprising 87 (36.86%) of the total. Figure 2 illustrates the nine most common molecular scaffolds that contain at least one ring in NPDBEjeCol, which collectively encompass over 30% of the 236 compounds present in the database. The molecular scaffolds that remain account for a proportion of less than 1.27% of the total. This quantity is equivalent to three molecules, with the base scaffold serving as the fundamental structural scaffold. The number and percentage of each scaffold in NPDBEjeCol is indicated below the chemical structures.

As illustrated in Figure 2, the presence of six-membered carbocycles is apparent, with benzene being represented in three distinct scaffolds. Benzene is the most common core scaffold in chemical databases used in drug discovery.^39^ The observed value for benzene scaffolds (10.59%) is analogous to that of BIOFACQUIM (9.7%). Benzene derivatives play an important role in a wide range of plant natural products; these compounds are fundamental to natural product chemistry due to their ability to interact with a variety of biological systems.^40^ Another noteworthy scaffold in NPDBEjeCol is cyclohexane, which accounts for 4.24% of the total. Additionally, three distinct scaffolds contain cyclohexanes. This cycle is also common in other databases, such as FooDB (2%).^41^ Cyclohexane is a structure found in a wide variety of natural products derived from plants, particularly in compounds such as terpenoids, alkaloids, fatty acids, phytosterols and some phenolic compounds. Cyclohexane and its derivatives are important in natural product chemistry because of their physical and biological properties and their ability to form part of more complex structures.^42^ Other notable scaffolds include xanthones, sterane derivatives, and chromane rings, the latter of which is particularly prevalent in bioactive compounds.^43^

### 3.6 Fragments Diversity

**Figure 3:**
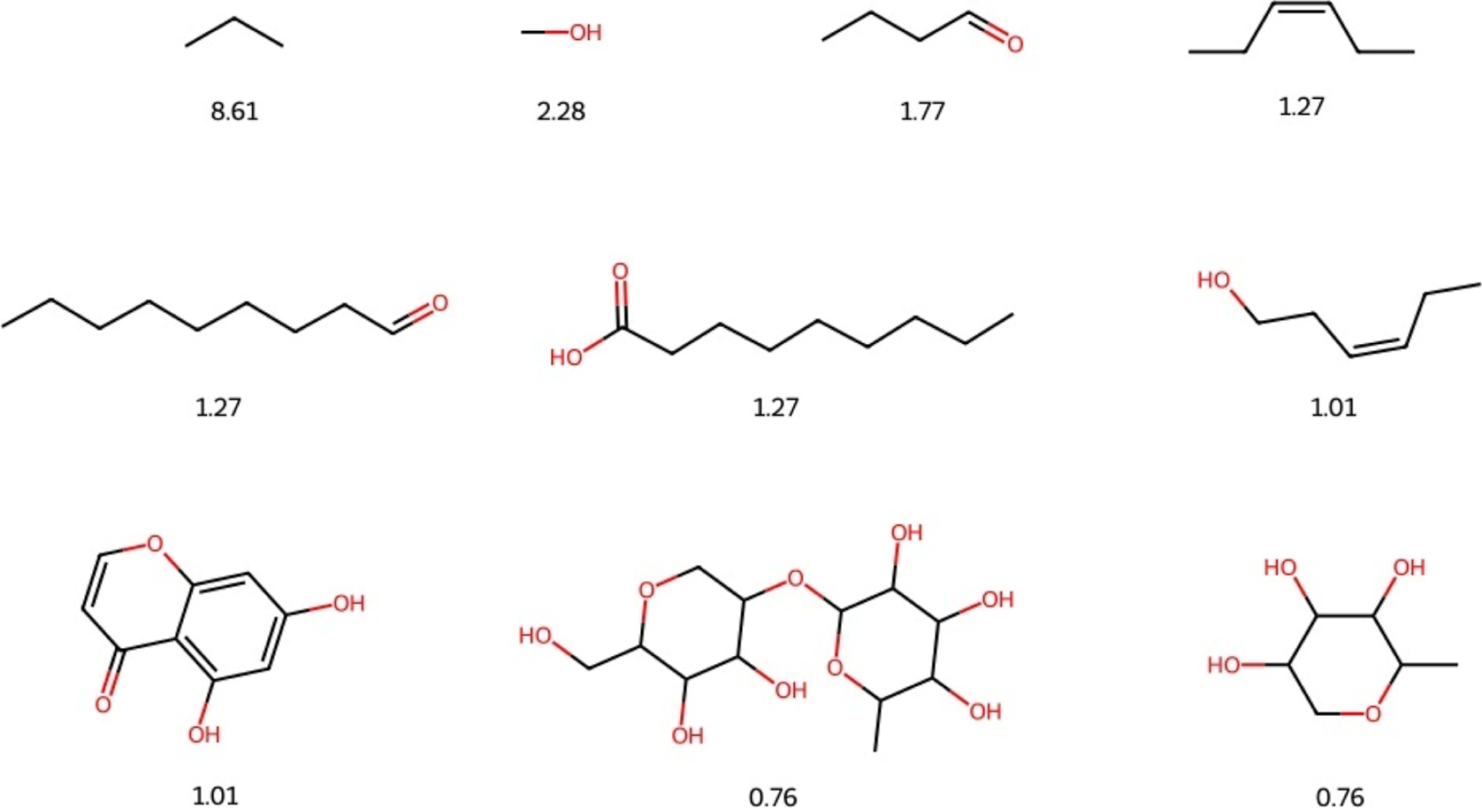
Ten most frequent fragments from NPDBEjeCol. The percentage of each fragment within its database is indicated below the chemical structures.

Figure 3 shows the number of fragments generated for NPDBEjeCol, along with the corresponding percentage for each. Of the 236 molecules, 231 exhibited fragmentation, while the remaining four demonstrated a MW exceeding 1000 g/mol.

A total of 200 fragments were obtained after applying the RECAP algorithm. In the case of NPDBEjeCol, the most abundant fragment (8.61%) is a three-member carbon chain, followed by a fragment with an abundance of 2.28%, which is a carbon-bearing hydroxyl group. Seven of the fragments are linear chains with one to nine carbons exhibiting terminal carbonyl and carboxyl groups. Additionally, two of these fragments display carbon–carbon double bonds. Three of the fragments correspond to rings, with heterocycles predominating and all containing oxygen. A peer-reviewed research article presents a comparative analysis of the unique fragment diversity of NPDBEjeCol with that of other natural product databases.^44^

### 3.7 Fingerprint-Based Diversity

**Figure 4:**
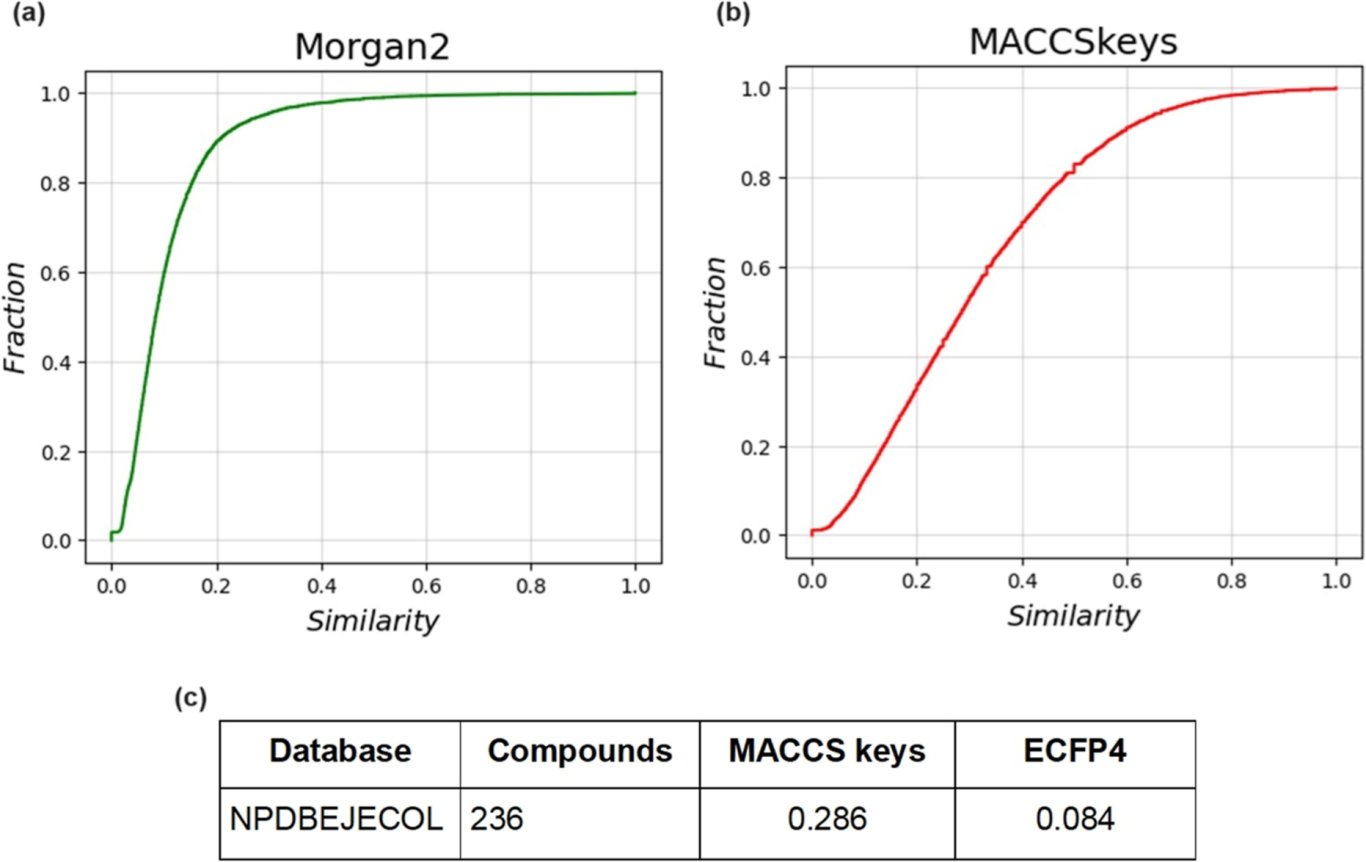
Cumulative distribution functions of the pairwise Tanimoto similarity. (a) Morgan2 fingerprint. (b) MACCS keys (166 bits) fingerprint. (c) The table summarizes the median value of the distributions.

**Figure 5:**
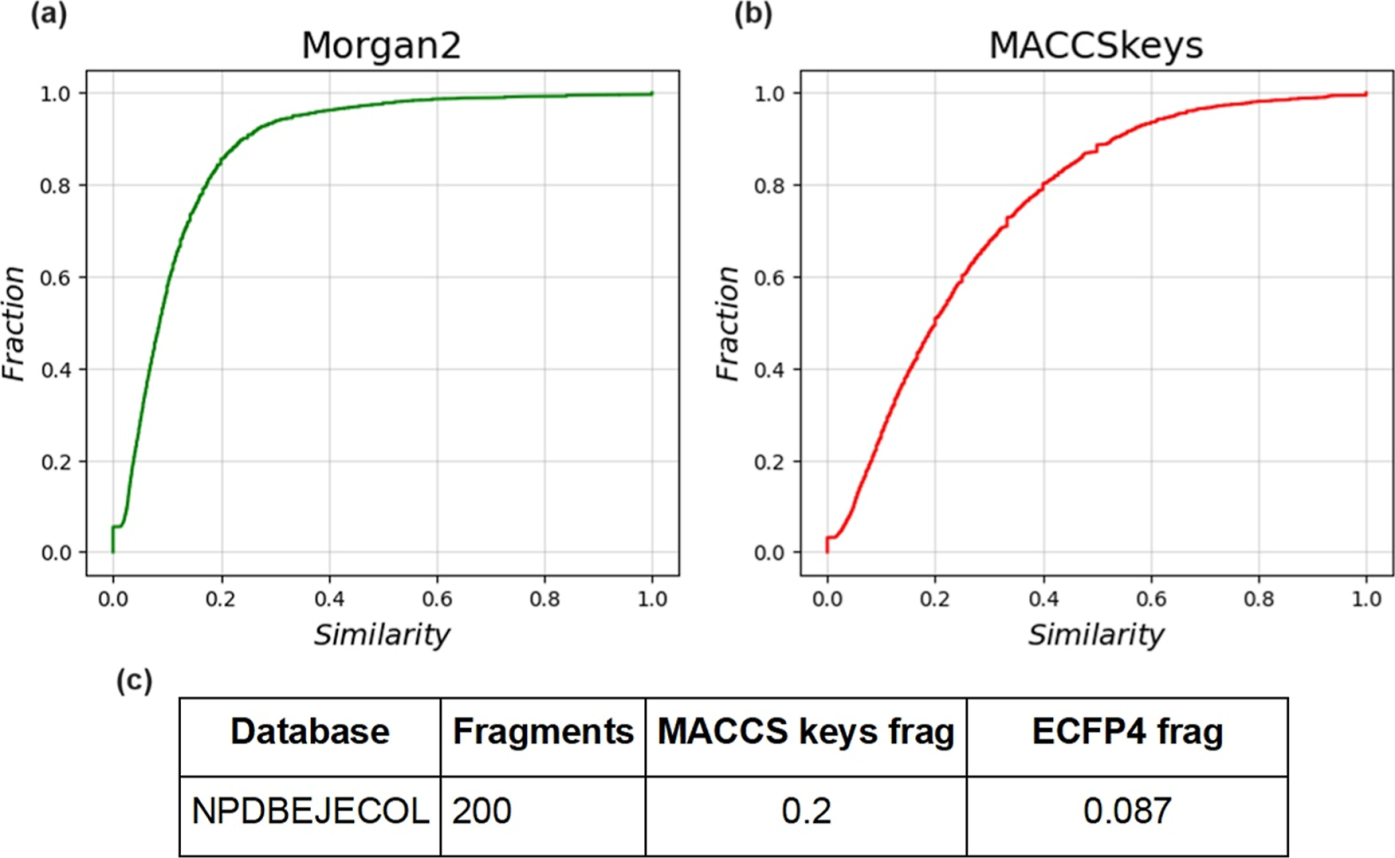
Cumulative distribution functions of the pairwise Tanimoto similarity. (a) Morgan2 fingerprint. (b) MACCS keys (166 bits) fingerprint. (c) The table summarizes the median value of the distributions.

The fingerprint-based structural diversity of the compound and fragment library was measured using cumulative distribution functions of the pairwise similarity values calculated with the Tanimoto coefficient and the Morgan2 and MACCS keys fingerprints. The results are presented in Figures 4 and 5. As illustrated in Figure 4, the chemical structures of NPDBEjeCol show an average similarity of 0.286 for MACCS keys and 0.084 for Morgan2. A comparison of these values with those reported by Chávez-Hernández et al.,^3^ reveals that, for MACCS keys, the value is lower than that of the databases under consideration. COCONUT (0.380), Food Database—FooDB (0.322), and DCM (0.136). The results for the Morgan2 fingerprint are similar, with the value for NPDBEjeCol being lower than those for COCONUT (0.107), FooDB (0.092), and DCM (0.136). These results indicate that NPDBEjeCol is the most diverse database as measured with the Tanimoto coefficient and both fingerprints.

Figure 5 shows the results for the fragment library obtained from NPDBEjeCol, exhibiting average similarity values of 0.200 for MACCS keys, and 0.087 for Morgan2. Chavez-Hernandez et al.,^3^ employed the same methodology as in this study. A comparison of the values revealed that the NPDBEjeCol fragments show greater structural diversity compared to the COCONUT, FooDB, and DCM fragments. The mean reference values are summarized in Table 2.

**Table 2:** Summary of Structural Diversity Based on Fingerprints^3^^,^^a^

data set of compounds	Morgan2 (1024 bits)	MACCS keys (166 bits)
COCONUT	0.107	0.380
FooDB	0.092	0.322
DCM	0.136	0.407

data set of fragments	Morgan2 (1024 bits)	MACCS keys (166 bits)
COCONUT	0.111	0.300
FooDB	0.106	0.241
DCM	0.125	0.243

a Mean value of the distribution.

### 3.8 Visual Representation of the Chemical Space

**Figure 6:**
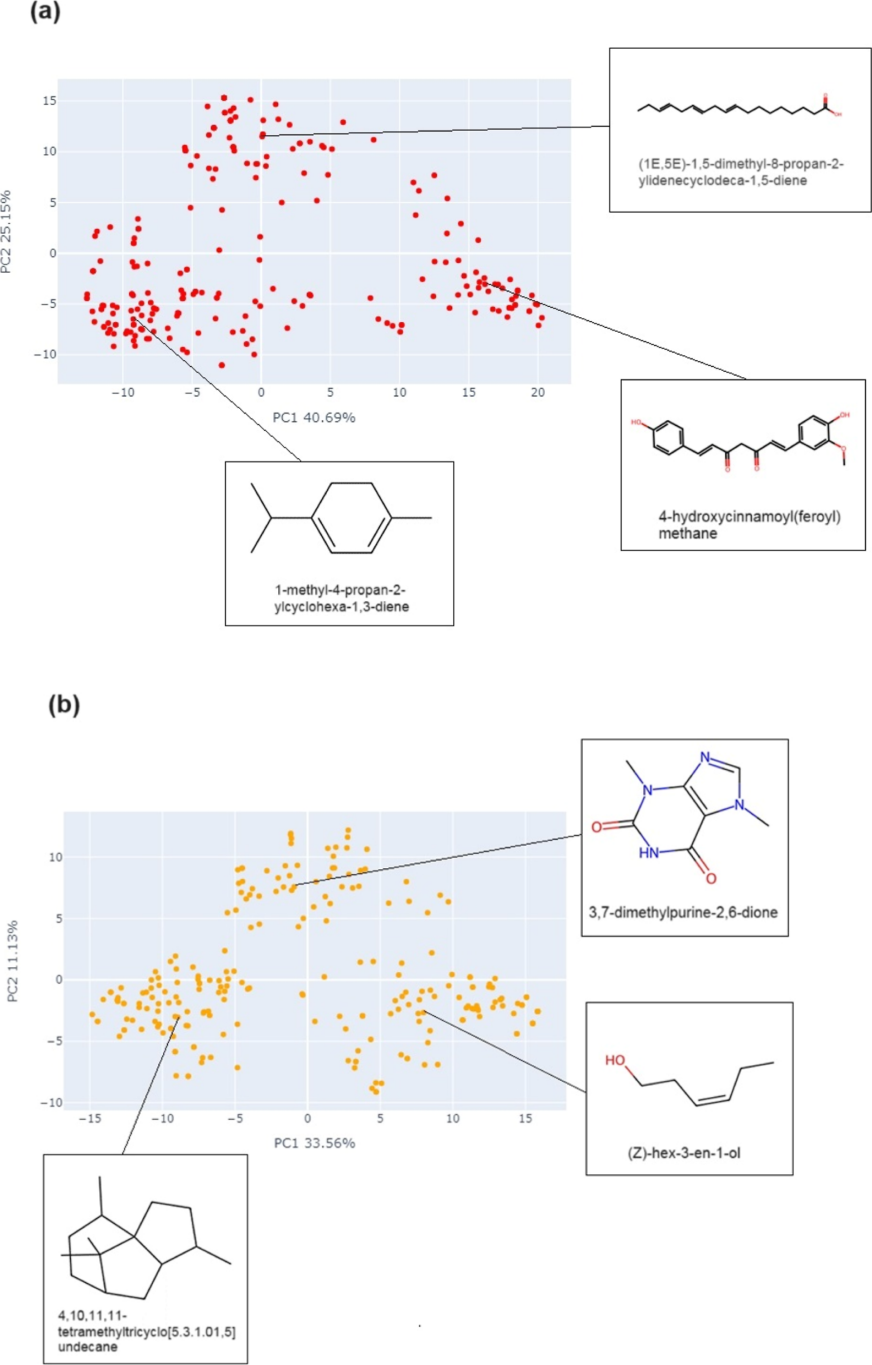
Chemical space visualization of the compounds from NPDBEjeCol using principal component analysis based on (a) MACCS keys and (b) Morgan2 fingerprints.

**Figure 7:**
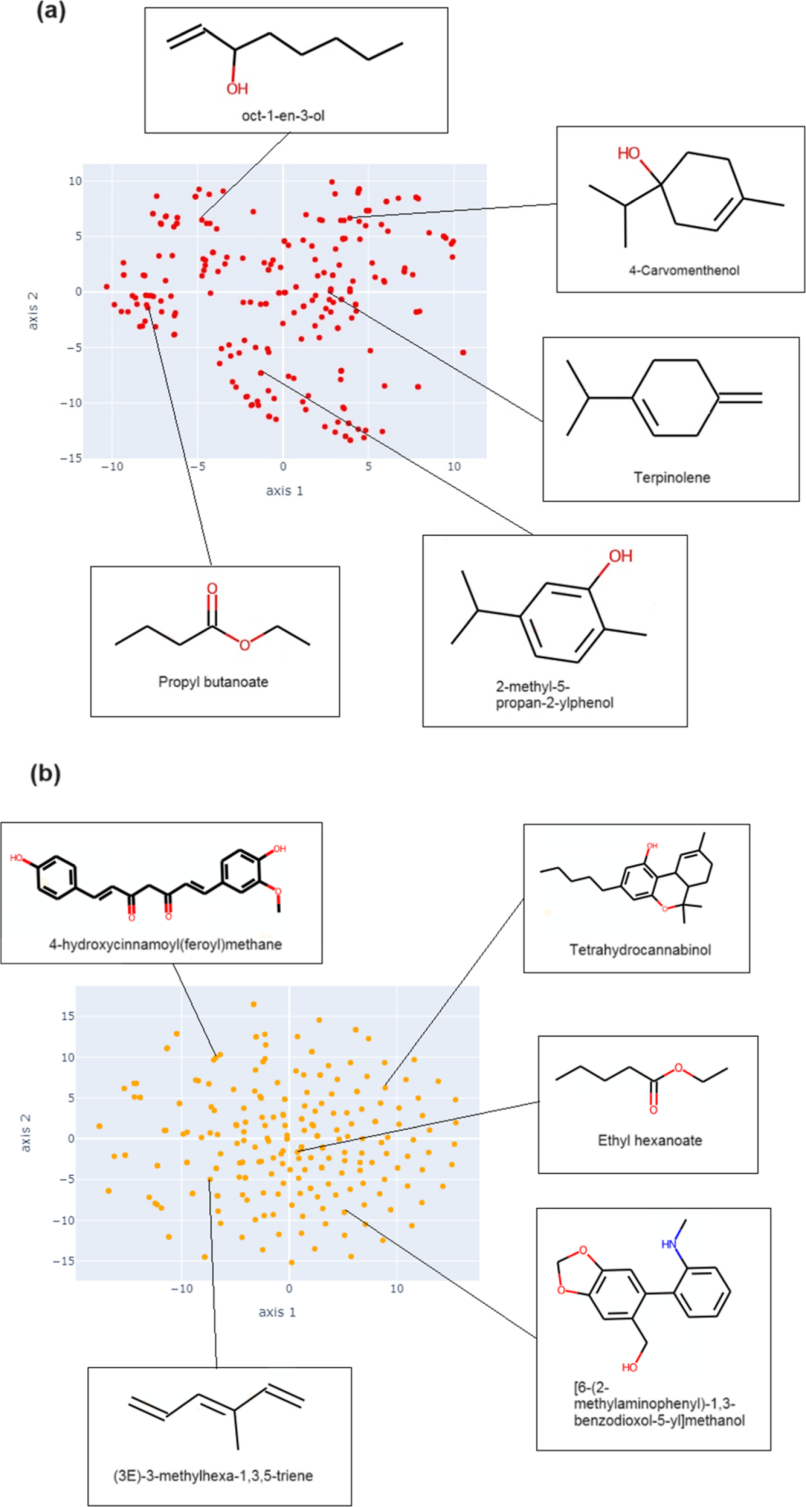
Chemical space visualization of the compounds from NPDBEjeCol using t-SNE based on (a) MACCS keys and (b) Morgan2 fingerprints.

**Figure 8:**
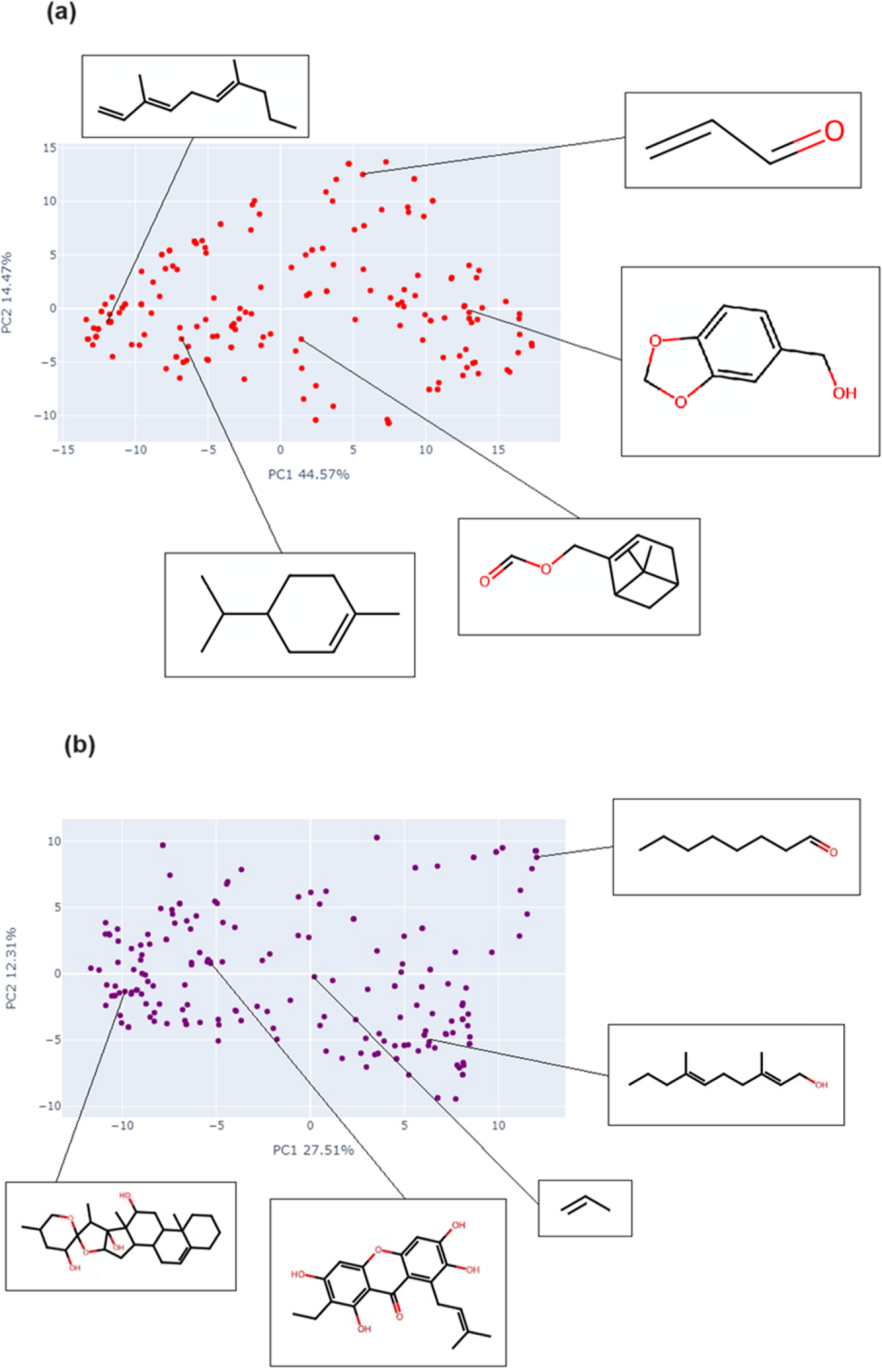
Chemical space visualization of the fragments compounds from NPDBEjeCol using principal component analysis based on (a) MACCS keys and (b) Morgan2 fingerprints.

**Figure 9:**
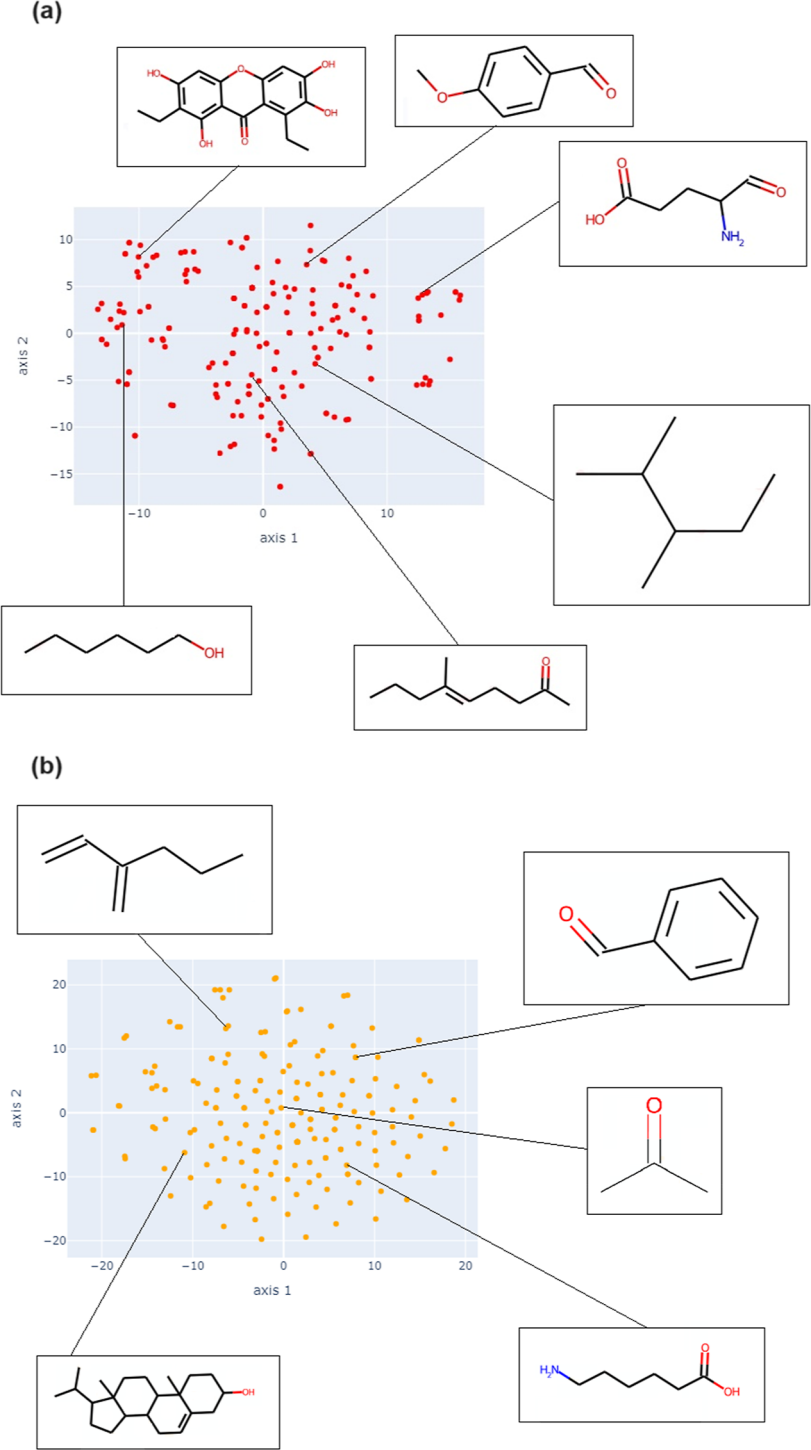
Chemical space visualization of the fragments compounds from NPDBEjeCol using t-SNE based on (a) MACCS keys and (b) Morgan2 fingerprints.

For the visual representation of the chemical space of NPDBEjeCol, two visualization methods, t-SNE and PCA, were used based on similarity matrices of pairwise comparisons calculated with the Tanimoto coefficient and two fingerprints with different design.^32^ Two similarity matrices were generated: one with 236 dimensions (Figures 6 and 7) representing the compounds, and a second with 200 dimensions (Figures 8 and 9) representing the fragments. In each case, examples of molecule structures in the identified clusters are shown. A peer-reviewed research article presents a comparative analysis of the chemical space of NPDBEjeCol with that of other natural products databases.^44^ To create visual representations of the chemical space of the NPDBEjeCol compounds and fragment library was performed as detailed in the Materials and Methods section.

The PCA for compounds (Figure 6) shows the representation of chemical space using MACCS Keys and Morgan2 fingerprints. For the MACCS Keys fingerprint, the first principal component (PC1) recovers 40.69% of the total variance in the data, while the second principal component (PC2) accounts for 25.10% (Figure 6a). For the Morgan2 fingerprint, the variance captured by the first and second principal components are 33.56% and 11.13%, respectively (Figure 6b). This evidence MACCS keys fingerprint represents a higher percentage of the total variance in the data for both components compared to Morgan2.

The PCA based on MACCS keys show three discrete groups of compounds: structures comprising carbocycles are situated within the bottom left group, other rings and heterocycles located within the right group, and branched linear molecules are located above the center (Figure 6a).

The PCA was utilized for the Morgan2 fingerprint, resulting in the identification of compounds within three distinct groups (see Figure 6b). The group located in the bottom left quadrant consists of molecules featuring fused rings and bridgeheads, in addition to groups comprising heteroatoms positioned outside the ring. The group that extends from the top toward the center is predominantly composed of rings containing heteroatoms. The third group, located at the bottom right, is made up of molecules with a predominantly linear structure, with or without functional groups containing heteroatoms.In terms of the visualization of the chemical space using t-SNE for the fingerprint of the MACCS keys, it is evident that the branched linear chains are located on the left side of the representation, while the cyclic compounds are distributed on the right.

Figure 7a highlights some examples. The situation differs somewhat when t-SNE is employed with the Morgan2 fingerprint. The distribution of molecules demonstrates a greater degree of dispersion. The center-left space is populated by branched linear molecules, while the remainder of the space is occupied by cyclic molecules. Figure 7b presents example molecules present in these regions. Similar to the PCA of compounds, the PCA of fragments (Figure 8) showed that the chemical space based on MACCS Keys captures a higher percentage of the total variance in the data for both components compared to Morgan2. For the MACCS Keys fingerprint, the first principal component (PC1) accounts for 44.57% of the total variance, while the second principal component (PC2) represents 14.47% (Figure 8a). In contrast, for the Morgan2 fingerprint, the first and second principal components capture 27.51 and 12.31% of the variance, respectively (Figure 8b).

The PCA employs the MACCS keys fingerprint to organize fragments into groups, wherein branched and linear molecules are predominant from top left to right, while different types of cycles are located from center to bottom left to right. Examples of fragments are shown in Figure 8a. In the case of the Morgan2 fingerprint, a dispersion of fragments is observed, with cycles located on the left and linear or branched chains are on the right (Figure 8b). With regard to the visualization of chemical space for the fragments using t-SNE for the MACCS keys fingerprint, the most linear fragments are distributed in the lower part of the chemical space, with the upper area reserved for cyclic fragments or those with cyclic portions (Figure 9a). Furthermore, visualization of the chemical space using t-SNE and Morgan2 fingerprinting demonstrates a more heterogeneous distribution of the fragments. The most linear fragments are situated in the center and at the extremes, while fragments with cyclic portions are dispersed throughout the chemical space (Figure 9b). An interactive version of chemical space visualization is freely available for download at https://github.com/rodrijohny/NPDBEjeCol.

### 3.9 Commercial Availability of Natural Products from NPDBEjeCol

The commercial availability of NPDBEjeCol natural products was evaluated using information available in the PubChem database. Of the 236 molecules in the library, each is commercially available.

### 3.10 NPDBEjeCol Design Web

A user interface was constructed to facilitate searching within the database, utilizing other natural product molecule databases, including COCONUT, BIOFACQUIM, PERUNPDB, and NuBBEDB, as references. The first version is freely available at https://npdbejecol.com/. The graphical user interface features a prominent search dialogue that allows users to search by various criteria, including line notation (SMILES, InChI key).

The project was implemented using a client-server infrastructure, in which an Apache server, a MySQL database engine and the PHP programming language were configured. The client-server architecture distributes tasks between two components: clients, which are devices such as computers or mobile phones that request information or services from the server, and servers, which are machines with greater processing and storage capacity, responsible for receiving, processing and sending the corresponding responses. Three applications were configured on the server: Apache, a web server that receives HTTP requests from users, processes the requests, and sends responses; MySQL, a relational database management system used to efficiently organize and retrieve data; and PHP, the programming language on which the Web site was programmed. The operation of the architecture is based on an interactive flow between client, server and the aforementioned technologies.

The basic search function enables the user to query the molecules stored in the database using specified search criteria, thereby returning a list of molecules that meet the condition. The information includes the molecular formula, number of C, N and O atoms and number of rings. The option to expand the search details for each molecule is available via a details button, which, when selected, displays the following elements: name, molecular formula, molecular weight, synonyms, total number of atoms, number of hydrogen acceptor and donor atoms, and a graphical representation of the molecule.

**Figure 10:**
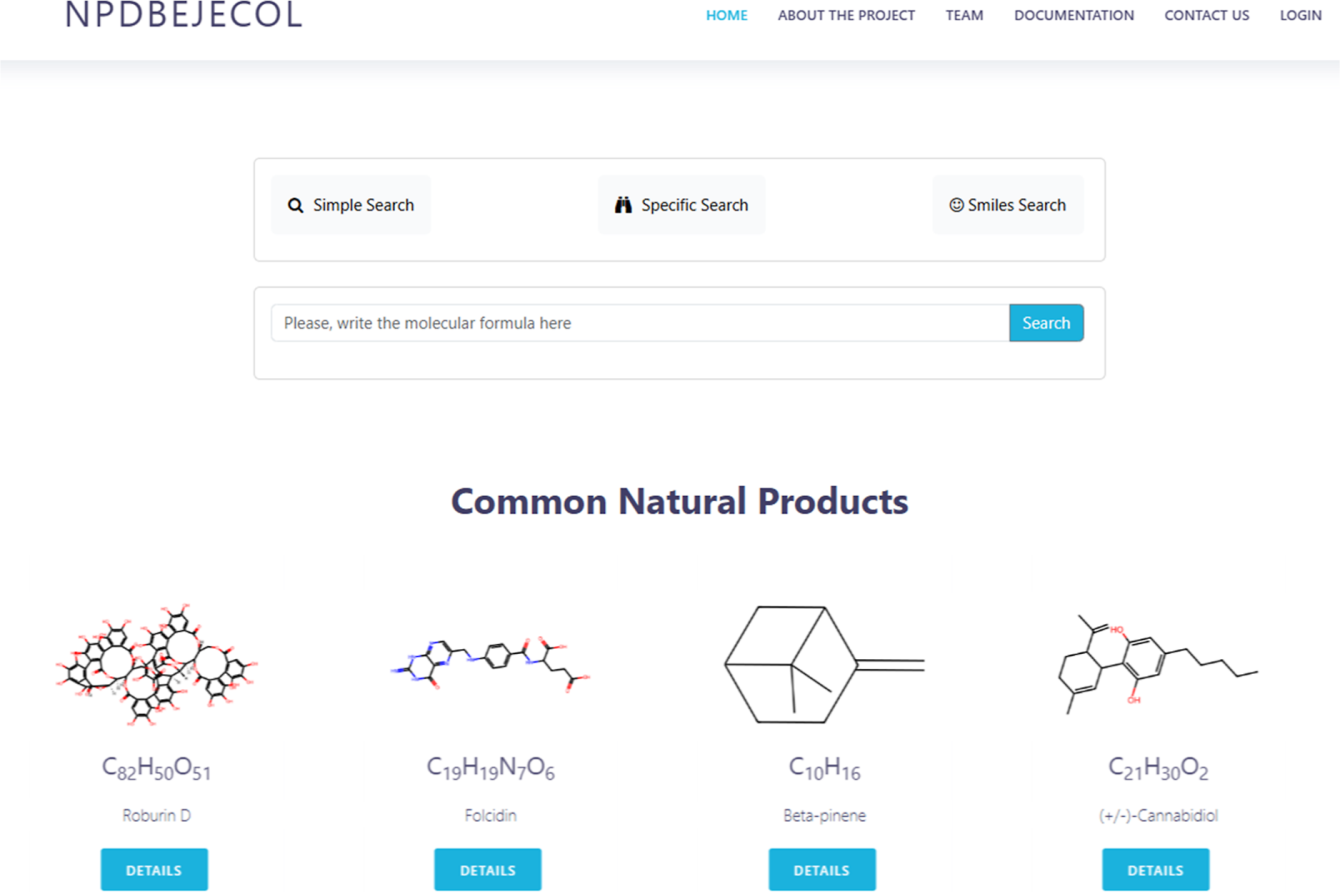
Homepage of the newly introduced database, https://npdbejecol.com/ (January 2025).

Additional search criteria include an advanced search with the following additional options: SMILES notation, InChI notation, InChI key, CAS number, IUPAC name, reference from which the molecule was taken, name of the plant from which the natural product comes, bond count, TPSA, LogP and calculated spectroscopic data by computational methods. Figure 10 shows the homepage of the site as of January 2025.

## 4 Conclusions

The NPDBEjeCol database was developed from a systematic review of data set compilation, specifically for the purpose of creating a proof of concept that exclusively covers the *coffee region* of Colombia. For each compound in the first version of the database the following information is included: identification number, compound name, SMILES, reference (journal name, DOI number, and year of publication), CAS number, synonym names and constitutional descriptors. The process of data set standardization revealed instances of duplicate molecules, which were removed, resulting in a data set of 236 molecules that can be freely downloaded in.csv format.

The calculated physicochemical properties of the compounds in NPDBEjeCol indicate an average MW of 234.77 g/mol, which is lower than that of comparable databases. The compounds have an average of three hydrogen bond acceptors (HBA) and one hydrogen bond donor (HBD). The average log *P* is 3.02 and the average TPSA is 47.72, consistent with Lipinski’s Rule of Five,^36^ indicating good absorption and permeability.^37^ NPDBEjeCol contains small molecules with an average of 14 carbon atoms, three oxygen atoms and minimal nitrogen content. Compared to other databases, NPDBEjeCol molecules have fewer atoms. Most molecules in it are linear structures, and the nine most common molecular scaffolds represent over 30% of the 236 compounds, with benzene being the most common, followed by cyclohexane. The major phytochemical groups in NPDBEjeCol are terpenes, fatty acids and derivatives of phenolic acids, alkaloids, pseudoalkaloids, flavonoids, and phenylpropanoids.

The RECAP algorithm yielded 200 fragments from 231 molecules, with the most prevalent fragment being a three-member carbon chain. The second most prevalent fragment is a carbon atom bearing a hydroxyl group. The predominant structural motifs among the fragments are linear chains and heterocycles containing oxygen. The structural diversity of the compounds and fragments was quantified using Tanimoto similarity with Morgan2 and MACCS keys fingerprints, revealing that the NPDBEjeCol set of molecules exhibited the greatest structural diversity. The mean similarity for MACCS keys was found to be lower than that observed for other comparable databases, including COCONUT, FooDB, and DCM. The mean similarity was also lower for Morgan2 in comparison with these databases. The fragment library also displays comparable trends, exhibiting a higher degree of diversity than other databases.

The visualizations of chemical space based on different fingerprints showed the existence of discrete clusters of compounds and fragments, which are delineated based on MACCS keys and Morgan2 fingerprints. The visualization illustrates the presence of discernible groupings of different structural types, with cyclic and linear molecules exhibiting distinct clustering patterns.

A review of the PubChem database reveals that all 236 molecules present in NPDBEjCol are commercially available.

## 5 Perspectives

In the future, NPDBEjeCol aims to establish itself as a comprehensive database of natural products, encompassing a greater number of molecules derived from a diverse range of natural sources, in addition to those derived from plants. Furthermore, it plans to expand its scope by incorporating data from other regions within Colombia. As a proof of concept, it is evident that a database of natural products for Colombia has a significant role to reveal the country’s potential in terms of this type of molecules, which is further enhanced by its status as a country with extensive biodiversity. As happens with other natural products collections, NPDBEjeCol has the potential to be used not only in drug discovery projects but in other research areas such as cosmetics where natural products also have a distinct role.

In terms of information curation, it is imperative to continuously refine molecular curation protocols to rectify any inaccuracies that may arise in the various published versions as the number of molecules increases. Moreover, it may be advantageous to consider expanding the set of molecular descriptors utilized to characterize each molecule, potentially incorporating additional new analysis categories.

A protocol for the web portal’s sustainability and periodic updating is planned, with consideration given to feedback from NPDBEjeCol users regarding the use and application of the database’s resources. It is our hope that NPDBEjeCol will not only maintain its functionality over time, but also become the reference database for natural products in Colombia.
